# Systolic blood pressure but not electrocardiogram QRS duration is associated with heart rate variability (HRV): a cross-sectional study in rural Australian non-diabetics

**DOI:** 10.1186/s40885-017-0065-1

**Published:** 2017-05-02

**Authors:** Yvonne Yin Leng Lee, Herbert F. Jelinek, Craig S. McLachlan

**Affiliations:** 1 Rural Clinical School, Samuels Building, Level 3, Faculty of Medicine, University of New South Wales, Sydney, NSW 2052 Australia; 2 School of Community Health, Charles Sturt University, Albury, Australia

**Keywords:** Electrocardiogram, HRV, Systolic blood pressure, Conduction

## Abstract

**Background:**

A positive correlation between ECG derived QRS duration and heart rate variability (HRV) parameters had previously been reported in young healthy adults. We note this study used a narrow QRS duration range, and did not adjust for systolic blood pressure. Our aims are to investigate associations between systolic blood pressure (SBP), QRS duration and HRV in a rural aging population.

**Methods:**

A retrospective cross sectional population was obtained from the CSU Diabetes Screening Research Initiative data base where 200 participants had no diabetes or pre-diabetes. SBP data were matched with ECG derived QRS duration and HRV parameters. HRV parameters were calculated from R-R intervals. Resting 12-lead electrocardiograms were obtained from each subject using a Welch Allyn PC-Based ECG system.

**Results:**

Pearson correlation analysis revealed no statistically significant associations between HRV parameters and QRS duration. No significant mean differences in HRV parameter subgroups across defined QRS cut-offs were found. SBP > 146 mmHg was associated with increasing QRS durations, however this association disappeared once models were adjusted for age and gender. SBP was also significantly associated with a number of HRV parameters using Pearson correlation analysis, including high frequency (HF) (*p* < 0.05), HFln (*p* < 0.02), RMSDD (*p* < 0.02) and non-linear parameters; ApEN (*p* < 0.001) were negatively correlated with increasing SBP while the low frequency to high frequency ratio (LF/HF) increased with increasing SBP (*p* < 0.03).

**Conclusions:**

Our results do not support associations between ECG derived R-R derived HRV parameters and QRS duration in aging populations. We suggest that ventricular conduction as determined by QRS duration is independent of variations in SA-node heart rate variability.

## Background

Activation of the Sinoatrial (S-A) node results in excitation of the atrio-ventricular (A-V) node [1]. Following which the impulse conduction is via the bundle of HIS conduction system and into the ventricular myocardium. Ventricular activation is responsible for the generation of the ECG derived QRS complex [2]. S-A node activation and cardiac autonomic contributions to the SA-node are typically studied via electrocardiogram (ECG) derived R–R intervals (around a mean value), using heart rate variability (HRV) [3, 4]. QRS duration during aging is typically associated with some widening of the ECG waveform due to conduction slowing; with aging there is also a reduction in HRV and an increase in aortic stiffening [5, 6]. Whether there are interactions between these physiological changes with aging has not been previously explored in a community setting.

Interestingly Nakagawa and associates reported a linear association between changes in QRS duration and HRV parameters, by assessing 24 h circadian changes in HRV and QRS duration [7]. We note that the QRS duration measures showing a linear correlation were greater than 100 msec indicating a possible association with increased cardiac ventricular mass or other conduction abnormalities. Unfortunately this study did not explore interactions with blood pressure. This study by Nakagawa et al [7] raises two important observations. Given that the S-A node is responsible for R-R HRV assessments as noted above, we wonder how this could be associated with ventricular conduction (QRS duration) via the A-V node [8] and whether aging would have an effect. On the other hand there is the theoretical possibility that increased sympathetic drive interacts with chronic blood pressure [9] to induce structural ventricular mass changes that may augment ventricular conduction slowing and an increase in QRS duration. Such interactions with systolic blood pressure (SBP) and sympathetic drive are complex, somewhat age dependent and relatively un-explored using HRV parameters [9].

Under conditions where there is increased isolated systolic blood pressure in aging populations, one may also expect an increase in QRS duration [6]. QRS duration >120 msec has been used as a bio-marker for increased cardiac risk in large epidemiological studies such as the Framingham Off-Spring Study [10]. We have not observed any studies to date that have examined the interactions of both QRS duration and systolic blood pressure on HRV parameters in a community cross-sectional study. Our aims are to investigate whether there is an HRV association with QRS duration in a rural population without diabetes or pre-diabetes. Diabetes and pre-diabetes are known to depress the cardiac parasympathetic autonomic nervous system - hence we have excluded this population from our analysis. Additional aims are to explore associations between systolic blood pressure and HRV.

## Methods

Ethics was obtained from Charles Sturt university human ethics committee (protocol approval number 2006/042). Written informed consent has been previously obtained from each recruited subject.

### Study population

Participants in this retrospective study were from the regional area of Albury-Wodonga and surrounding districts on the New South Wales-Victoria border in southeast Australia [6]. Data were drawn from the Charles Sturt University Diabetes Complication Screening Program data base containing 845 participants. A total of 554 participants had ECG QRS duration data and were in sinus rhythm. The sample size was further reduced to 200 participants that had matching HRV and who have been screened and met the criteria being non-diabetic. All participants were retrospectively selected from August 1, 2002 till September 14, 2012, for cross-sectional re-analysis.

Participants were required to complete a standardized questionnaire to ascertain presence of risk factors. They were asked about previous and current medical conditions such as diabetes or cardiovascular disease. Alcohol consumption and smoking were defined based on a yes or no questionnaire response, as completed by the patient. A response of ‘yes’ to smoking meant the patient smoked more than five cigarettes per day. A response of ‘yes’ to alcohol consumption meant the patient consumed more than two to three glasses of alcohol per day. Participants were classified as hypertensive if they had a known history of hypertension and/or a blood pressure measurement that was classified as hypertensive (greater than 140/90 mmHg). Subjects with no known diabetes or a random fasting blood glucose level <5.6 mmol/L were included if they had both an ECG with QRS duration and HRV data available.

### Weight and blood pressure assessments

Participants were asked to be in a relaxed supine position for 5 min prior to blood pressure (BP) measurement. The arm is at the level of the heart with the palm up for brachial measurement. Blood pressure cuff placed 2.5 cm above the site of pulsation with arrows centered where marked on cuff over the artery. The systolic and diastolic pressure value read off the automatic Welsh Allyn digital blood pressure monitor. Weight recorded in kilograms (kg) using generic scales while height is recorded in meters (m) using a stature meter. Body mass index (BMI) is derived from dividing weight (kg) over square of height (m^2^).

### Biochemical assessments

Whole blood samples were drawn from participant’s vein into two 7 ml EDTA tubes, one 10 ml with 100ul preservative heparin tube and one 7 ml plain tube. The blood samples are gently inverted for 8 to 10 times to ensure proper mixing in the prefilled EDTA and heparin tubes. High density lipoprotein levels were measured using plasma samples. High density lipoprotein was determined by immunoinhibition assay while low density cholesterol was calculated according to Friedewald formula [11]. Fasting blood capillary glucose measured using Accu-Check® system (Roche Australia Pty Limited). Total cholesterol and triglycerides tested using point-of-care instrument (BioRad P/L).

### 12-lead electrocardiogram

ECGs were acquired by either a Welch Allyn CardioPerfect device with software to auto-calculate QRS duration or or via a MacLab (ADInstruments, Australia) with an ECG bioamp, where signals were stored in Macintosh Chart version 5 with a sampling rate at 400Hz after applying a digital notch filter at 50Hz to reduce power-line interferences. MacLab acquired ECG parameters such as peak of R wave, peak of T wave and QRS duration were identified through an extraction algorithm using MLS310 HRV module version 1.0 (ADInstruments, Australia).

### HRV measurement

ECG signals were recorded for 20 min in the resting supine position. Raw digital ECG signals were first manually edited to remove any noise and ectopic beats. HRV was analyzed using the program Soft-ECG (copyright Herbert Jelinek). Before the ECG recording was converted into Soft-ECG format, the digital ECG trace was manually edited to remove any movement artifacts and ectopic beats [12]. Soft-ECG converts the raw ECG trace into an R-R interval graph for analysis. R-R intervals are determined by using the criteria for detecting the fiducial point of the QRS wave [12]. Once converted into an R-R graph, further intervals greater and smaller than 200 ms from the mean interval length were removed, as these were deemed to reflect ectopic beats or noise. The HRV parameters calculated include frequency domain analysis, time domain analysis, and nonlinear analysis measures [12, 13].

### Statistical analysis

Exploratory and descriptive analyses were performed. Sampling distribution of categorical variables was examined using chi-square test and *t*-test was used for continuous variables. Results were reported as mean and standard deviation for continuous data and proportions for discrete data. QRS duration were categorized into three groups as these QRS cut off values have shown prognostic significance; 80–100 msec; >100–120 msec; and >120 msec [6]. Exploratory associations between QRS duration and HRV parameters were assessed by Pearson correlation coefficient. Sensitivity analysis was performed to test the effects of HRV parameters with and without outliers on QRS duration (categorical and continuous). P value <0.05 were considered statistically significant. All analyses were conducted using STATA 13 software (StataCorp. 2013. *Stata Statistical Software: Release 13*. College Station, TX: StataCorp LP.)

## Results

### QRS duration and HRV

Summary population characteristics and demographics are presented in Table 1 across QRS durations. Female gender accounted for 60.9% of the study cohort. 16.3% of participants self-reported a pre-existing cardiovascular condition and 53.5% self-reported that they were hypertensive and being treated. We observed significant proportional increase in age, male gender and hypertension and preexisting CVD with increasing QRS durations (Table 1).

**Table 1:** Baseline characteristics of study cohort across three QRS groups

	QRS duration (ms)						*p*-value
	80–100 (*n* = 115)		>100–120 (*n* = 69)		>120 (*n* = 16)		
	n	%	n	%	n	%	
Age (years), mean (SD)	65.5 (11.2)		67.4 (10.1)		72.7 (6.8)		0.032
Gender							0.002
Female	82	71.3	31	44.9	9	56.3	
Male	33	28.7	38	55.1	7	43.8	
BMI	27 (5.2)		28.4 (4.4)		26.3 (2.3)		0.091
Hypertension	44	38.3	40	58	10	62.5	0.015
Cardiovascular	17	14.8	12	17.4	4	25	0.57
Smoking	2	1.7	1	1.5	0		0.87
Alcohol	5	4.4	5	7.3	1	6.3	0.70
Exercise	6.7 (3.4)		6.5 (3.3)		7.5 (3.3)		0.61
SBP	127.1 (17.7)		131.1 (15.7)		127.2 (16.2)		0.30
DBP	75.4 (8.1)		76.8 (8.2)		76.7 (6.8)		0.50
Total cholesterol	5.3 (1.1)		5.3 (1.0)		5.1 (0.9)		0.87
LDL	3.1 (1.0)		3.2 (0.8)		3.0 (0.9)		0.86
HDL	1.6 (0.5)		1.6 (0.5)		1.5 (0.3)		0.53
Triglycerides	1.2 (0.7)		1.1 (0.5)		1.4 (0.5)		0.38
Creatinine	67 (15.8)		71.6 (19.9)		65.6 (14.5)		0.20

*SD* standard deviation, *BMI* body mass index, *SBP* systolic blood pressure, *DBP* diastolic blood pressure, *LDL* low density lipoprotein, *HDL* high density lipoprotein

We performed sensitivity analysis on HRV parameter outliers. Outliers were identified using boxplot methods. Outliers were defined as being 1.5 times greater than the interquartile range beyond the boxplot range [14, 15]. Sensitivity analysis showed that HRV outliers had no influence on the association between HRV parameters and QRS duration. Table 2 demonstrates the distribution of HRV domains (without outliers); frequency domain, time domain and nonlinear analysis parameters, against three QRS subgroups based on QRS duration ranges (80–100 ms (normal), 101–120 ms (increased risk for cardiac mass) and >120 ms (likely increase in cardiac mass). On the basis of stratifying QRS duration, no significant mean differences were noted across the different HRV parameter sub-groups. We further assessed the relationship between HRV and QRS duration using Pearson correlation, similarly no significant associations in HRV and QRS duration were observed. Further stratification of QRS duration, using cutoff’s greater than 140 ms and 150 ms, also revealed no differences across mean HRV parameters using these additional arbitrary QRS duration cutoffs. In the subsequent subgroup analysis, we excluded subjects with self-reported hypertension and cardiovascular conditions (Table 3). The association between QRS and HRV, similarly non-significant interactions are reported.

**Table 2:** Heart rate variability parameters; frequency, linear and non-linear, across three different QRS groups

			QRS duration (ms)				*p*-value
	80–100 (*n* = 115)		>100–120 (*n* = 69)		>120 (*n* = 16)		
	n	Mean (SD)	n	Mean (SD)	n	Mean (SD)	
Frequency domain, mean (SD)							
Total power (ms^2^)	102	1222.9 (781.7)	62	1302.4 (793.9)	14	1233.3 (591.7)	0.81
HF (ms^2^)	92	192.2 (134.7)	59	181.7 (125.3)	14	2229.8 (145)	0.47
LF (ms^2^)	95	264.9 (178)	62	300.4 (194.7)	15	312.6 (229.4)	0.42
HF In	100	5.3 (1.1)	52	5.3 (1.0)	11	5.6 (0.8)	0.73
LF In	99	5.6 (0.9)	52	5.7 (0.8)	11	5.9 (0.9)	0.59
HF norm (v)	115	44.3 (18.2)	69	42.2 (18.1)	15	42.3 (23.0)	0.73
LF norm (v)	115	55.1 (18.1)	68	58.1 (18.5)	16	51.5 (23.3)	0.37
LF/HF ratio	101	1.3 (0.8)	60	1.5 (0.9)	13	1.2 (0.9)	0.36
Time domain, mean (SD)							
SDNN (ms)	108	38.9 (13.7)	65	38.9 (13.2)	14	38.7 (12)	1.00
RMSDD (ms)	103	25.7 (10.4)	63	24.7 (10.3)	15	29.1 (11.8)	0.34
Nonlinear analysis							
DFA α1	115	1.0 (0.2)	69	1.1 (0.3)	16	1.1 (0.4)	0.43
DFA α2	111	1.0 (0.2)	67	1.0 (0.2)	16	0.9 (0.2)	0.60
DFA 32	0		0		0		
ApEN	110	1.3 (0.2)	69	1.2 (0.2)	16	1.2 (0.2)	0.093

*SD* standard deviation, *HF* high frequency, *LF* low frequency, *norm* normalized, *SDNN* standard deviation of R-R intervals, *RMSDD* root mean square of successive difference of RR, *DFA* detrended fluctuation analysis, *ApEN* approximate entropy

**Table 3:** Frequency, time and nonlinear domain analysis of heart rate variability parameters across QRS groups (non-diabetes, no history of hypertension and no self reported cardiovascular conditions)

	QRS duration (ms)						*p*-value
	80–100 (*n* = 66)		>100–120 (*n* = 26)		>120 (*n* = 4)		
	n	mean (SD)	n	mean (SD)	n	mean (SD)	
Frequency domain, mean (SD)							
Total power (ms^2^)	61	1342.9 (833.9)	24	1280.8 (752.3)	4	1428.5 (446.3)	0.92
HF (ms^2^)	55	203.1 (130.3)	24	186.3 (133.2)	4	217.5 (114.7)	0.83
LF (ms^2^)	56	303.1 (194.8)	24	335.7 (192.4)	4	349.8 (255.6)	0.74
HF In	57	5.3 (1)	21	5.2 (0.9)	2	5.5 (0.5)	0.89
LF In	57	5.7 (0.9)	21	5.8 (0.7)	2	5.4 (1.3)	0.76
HF norm (v)	66	42.5 (15.6)	26	37.9 (18.4)	4	43 (27.2)	0.49
LF norm (v)	66	56.6 (15.5)	26	62.8 (18.9)	4	44.6 (11.3)	0.072
LF/HF ratio	60	1.4 (0.7)	22	1.8 (1.1)	3	0.9 (0.5)	0.059
Time domain, mean (SD)							
SDNN (ms)	64	39.8 (12.8)	26	39.3 (13.8)	4	46.5 (17)	0.59
RMSDD (ms)	61	25.7 (9)	25	23.5 (7.9)	3	27.4 (8.3)	0.52
Nonlinear analysis							
DFA α1	66	1.1 (0.2)	26	1.2 (0.2)	4	1.1 (0.3)	0.12
DFA α2	64	1.0 (0.2)	26	1.0 (0.2)	4	0.9 (0.1)	0.29
DFA32	0		0		0		
ApEN	65	1.3 (0.1)	26	1.2 (0.2)	4	1.2 (0.1)	0.19

*SD* standard deviation, *HF* high frequency, *LF* low frequency, *norm* normalized, *SDNN* standard deviation of R-R intervals, *RMSDD* root mean square of successive difference of RR, *DFA* detrended fluctuation analysis, *ApEN* approximate entropy

We next investigated the relation between SBP and HRV, as sympathetic drive influencing SBP may also influence HRV and QRS interactions (Table 4). SBP measures were stratified according to their natural quartiles. This allowed for preliminary exploratory assessment to assess any associations between SBP and HRV. We found that at least one parameter from each of the HRV domains was significantly related to SBP (Table 4). HRV frequency domain; HF (*r* = 0.16, *p* < 0.05), LF (0.017), time domain; RMSDD (*r* = 0.19, *p* < 0.013) and non-linear parameters; ApEN (*r* = 0.29, *p* < 0.001) were negatively correlated with increasing SBP while LF/HF ratio increased with increasing SBP (*r* = 0.18, *p* = 0.026). The correlation between SBP and HRV after removal of hypertension and CVD, revealed a moderate significant association. In the linear regression analysis after adjusting for age in the multivariate model, the HRV parameter ApEn, remained significant for an interaction with SBP (Table 5).

**Table 4:** Frequency, time and nonlinear domain analysis of heart rate variability parameters across systolic blood pressure natural quartiles among healthy individuals (non-diabetes, hypertension and cardiovascular conditions)

Heart rate variability	Systolic blood pressure (mm Hg)								
	70–120 (*n* = 82)		121–130 (*n* = 43)		131–145 (*n* = 39)		146–220 (*n* = 14)		*p*-value
	n	Mean (SD)	n	Mean (SD)	n	Mean (SD)	n	Mean (SD)	
Frequency domain, mean (SD)									
Total power (ms^2^)	69	1282.4 (750.7)	38	1622.8 (843.6)	34	1046.5 (640.2)	13	1326 (810.5)	0.016
HF (ms^2^)	65	197.3 (135.1)	36	222.9 (130.5)	34	140.6 (108.1)	14	162.8 (136.2)	0.046
LF (ms^2^)	69	321.9 (203.4)	36	320.9 (190.2)	33	234 (166.8)	14	305.3 (214.3)	0.21
HF In	77	5.4 (1.1)	40	5.5 (0.9)	33	4.8 (1.1)	12	4.7 (1.0)	0.0064
LF In	78	5.8 (1.0)	40	5.8 (0.9)	32	5.5 (0.9)	12	5.6 (0.7)	0.54
HF norm (v)	81	40.6 (16.1)	43	42.1 (16)	39	38.3 (18.4)	14	31.4 (13.4)	0.17
LF norm (v)	82	57.1 (16.4)	43	55.2 (15.3)	39	60.2 (18.7)	14	66.8 (12.8)	0.10
LF/HF ratio	71	1.39 (0.7)	39	1.4 (0.8)	32	1.6 (0.9)	11	2.1 (1.0)	0.029
Time domain, mean (SD)									
SDNN (ms)	79	40.9 (14.1)	43	44.6 (13.1)	38	36.8 (13.1)	14	38.5 (13.9)	0.077
RMSDD (ms)	75	25.8 (10.5)	40	27 (8.9)	37	21.9 (9.8)	13	19.6 (8.9)	0.025
Nonlinear analysis									
DFA α1	82	1.1 (0.2)	43	1.0 (0.3)	39	1.1 (0.3)	14	1.1 (0.2)	0.55
DFA α2	82	0.9 (0.2)	43	1.0 (0.2)	36	1.0 (0.1)	14	1.0 (0.1)	0.68
DFA32	32	92.6 (31.5)	22	87.9 (30.9)	11	82.4 (31.1)	8	88 (27.2)	0.81
ApEN	81	1.26 (0.14)	39	1.18 (0.18)	36	1.16 (0.22)	12	1.06 (0.22)	0.001

*SD* standard deviation, *HF* high frequency, *LF* low frequency, *norm* normalized, *SDNN* standard deviation of R-R intervals, *RMSDD* root mean square of successive difference of RR, *DFA* detrended fluctuation analysis, *ApEN* approximate entropy

**Table 5:** Linear regression analysis between SBP and HRV (both as continuous variables) - among “healthy” individuals (without diabetes, hypertension or self reported cardiovascular conditions)

Heart rate variability			SBP	
	Univariate		Multivariate (adjusted for age)	
	Coefficient (*r*)	*p*-value	Coefficient (*r*)	*p*-value
Frequency domain, mean (SD)				
Total power (ms^2^)	−0.001	0.59	−0.001	0.67
HF (ms^2^)	−0.02	0.05	−0.015	0.13
LF (ms^2^)	−0.01	0.28	−0.043	0.51
HF In	−2.65	0.017	−1.67	0.12
LF In	−1.06	0.42	0.15	0.91
HF norm (v)	−0.97	0.18	−0.085	0.23
LF norm (v)	0.13	0.07	0.11	0.11
LF/HF ratio	3.52	0.026	2.79	0.064
Time domain, mean (SD)				
SDNN (ms)	−0.09	0.31	−0.029	0.74
RMSDD (ms)	−0.31	0.013	−0.23	0.051
Nonlinear analysis				
DFA α1	1.65	0.74	0.58	0.9
DFA α2	7.11	0.37	0.55	0.94
DFA32	−0.033	0.6	0.031	0.59
ApEN	−25.1	<0.001	−20.55	0.001

*HF* high frequency, *LF* low frequency, *norm* normalized, *SDNN* standard deviation of R-R intervals, *RMSDD* root mean square of successive difference of RR, *DFA* detrended fluctuation analysis, *ApEN* approximate entropy

With evidence that SBP interacted with HRV, we further explored QRS and HRV associations in a multivariate model adjusting for age, gender and SBP. Correspondingly, there was no significant difference in the QRS duration across any of the HRV parameters.

We further performed sensitivity analysis to examine the relationship between SBP and QRS duration. Using the entire cohort, highest SBP quartiles (146 mmHg to 220 mmHg) had a positive linear relationship with QRS duration (b = 3.63, *p* = 0.022). This indicates SBP >146 mmHg is associated with increasing QRS duration. However, this association was no longer significant after adjustment for age, gender, BMI, hypertension and cardiovascular.

## Discussion

Our main findings do not support the hypothesis that R-R derived HRV parameters are associated with increasing QRS duration in non-diabetics. The QRS duration cut offs that we modeled would reflect both normal and underlying conduction disturbances observed in rural communities [6, 16]. QRS duration greater than 120 msec in our cohort were most likely due to pre-existing cardiovascular disease, age related aortic stiffening, remodeling and or conduction slowing [6]. Indeed a significant proportion of the cohort had self reported hypertension. Our findings are in contrast with the results published by Nakagawa and colleagues [7]. This study by Nakagawa et al [7] reported that a QRS duration between 95 and 109 ms was inversely correlated with LF/HF ratio (*r* = 0.94, *p* < 0.001) and positively correlated with HF (*r* = 0.93, *p* < 0.001) in some subjects. When we replicated a similar QRS cut-off of >100 to 120 msec in our cohort and examined the association between HRV parameters LF/HF ratio and HF, we found no interaction between these parameters.

The reason for the lack of association with HRV parameters and QRS duration is likely because the cardiac autonomic nervous system contributions to the S-A node and A-V node are independent of one another. The S-A node is unlikely to contribute to ventricular repolarization (eg. QRS duration). Atria pacing studies have confirmed that changes in HRV are predominately derived from variations of the sinus node pulse formation [17–19] suggest that there are two distinct levels of vagal modulation, one at the sinus node and one at the ventricular level, both of which are different. This has been confirmed by ablation of the atria and observing the preservation of rate control in the ventricles, and via stimulation of the vagus nerve, inducing ERP prolongation at ventricular sites. Vagally induced prolongation of ventricular ERP is similar between denervated and intact sham-operated dogs, indicating independent and direct efferent vagal-ventricular innervations [17]. Interestingly studies of atrial fibrillation (AF) demonstrate ventricular HRV continues to behave as it is sinus rhythm [20]. Additionally carotid sinus nerve stimulation in atrial fibrillation increases vagal outflow and lowers ventricular rate [21]. In our studies with both normal and abnormal QRS durations it is not possible to observe associations with SA-nodal HRV.

We appreciate that the SA-node is influenced by the adjustments in cardiac autonomic balance from central or peripheral autonomic systems, such as afferent, central nuclei, efferent, and cardiac autonomic receptors [22]. These same systems may be also involved in blood pressure control. Long term development of chronic changes in systolic blood pressure are likely as a result of a mixture of hormonal, renal, aortic stiffening and neural influences [23, 24].

Interestingly, all HRV parameters were associated with increases in SBP. For example HRV frequency domain; HF, time domain; RMSDD and non-linear parameters; ApEN were negatively correlated with increasing SBP. While LF/HF ratio increased with increasing SBP. This suggests that changes in both sympathetic drive and parasympathetic changes were present with increasing SBP. We note the possibility of a reduction in parasympathetic drive with RMSDD decreasing with increasing SBP and possibly a decrease with the LF/HF ratio increasing. On the other hand when we adjusted for age, ApEn remained significant interacting with SBP. This confirms a previous observation where Schmitt and Ivanov [25] demonstrated that heart beat fluctuations underlying the coupled cascade of cardiac autonomic nonlinear and fractal feedback loops remain intact with advanced healthy aging. We cannot determine if the association of ApEn with increasing SBP is due to changes in vagal withdrawal and or increases in sympathetic drive [26].

In our study the mean SBP measures across QRS cut-offs were not significantly different. We did note SBP >146mmg were associated with higher QRS cut-offs in unadjusted models. The mean SBP levels in the community population were high, likely a reflection of aging populations. An association of higher SBP and increased QRS duration would be suggestive of cardiac hypertrophy [6]. However, in a previous study we noted the absence of elevated systolic aortic pressure to be associated with QRS durations >120 msec [6]. This too may suggest why SBP was not associated with QRS duration in our aging rural population.

Limitations of this study are that our study design is cross-sectional and therefore we can not imply causality from association studies. We did not have data on medications that may have influenced cardiovascular parameters in this study, on the other hand we also appreciate that in rural areas of Australia blood pressure medication compliance is low and that blood pressure is under diagnosed [27].

## Conclusions

Our results do not support associations between ECG derived R-R derived HRV parameters and QRS duration in aging populations. We suggest that ventricular conduction as determined by QRS duration is independent of variations in SA-node heart rate variability. HRV would not be useful to assess cardiac hypertrophy interventions. On the other hand one could theoretically anticipate an improvement in HRV parameters with improvements in elevated systolic blood pressure.

## Abbreviations

ApEN: Approximate entropy
AV: Atrio-ventricular
BMI: Body mass index
BP: Blood pressure
CVD: Cardiovascular disease
ECG: Electrocardiogram
HF: High frequency
HRV: Heart rate variability
LF: Low frequency
RMSSD: Square root of the mean squared differences of successive NN intervals
SA: Sinoatrial
SBP: Systolic blood pressure
